# Uridine 5′-Triphosphate Promotes *In Vitro* Schwannoma Cell Migration through Matrix Metalloproteinase-2 Activation

**DOI:** 10.1371/journal.pone.0098998

**Published:** 2014-06-06

**Authors:** Aloa Lamarca, Alejandro Gella, Tania Martiañez, Mònica Segura, Joana Figueiro-Silva, Carmen Grijota-Martinez, Ramón Trullas, Núria Casals

**Affiliations:** 1 Department of Basic Sciences, Facultat de Medicina, Universitat Internacional de Catalunya, Sant Cugat del Vallès, Spain; 2 Neurobiology Unit, Institut d′Investigacions Biomèdiques de Barcelona, Consejo Superior de Investigaciones Científicas, Institut d′Investigacions Biomèdiques Pi i Sunyer, Barcelona, Spain; 3 Centro de Investigación Biomédica en Red de Fisiopatología de la Obesidad y Nutrición (CIBERobn), Instituto de Salud Carlos III, Madrid, Spain; NCMLS, Radboud University Nijmegen Medical Center, Netherlands

## Abstract

In response to peripheral nerve injury, Schwann cells adopt a migratory phenotype and modify the extracellular matrix to make it permissive for cell migration and axonal re-growth. Uridine 5′-triphosphate (UTP) and other nucleotides are released during nerve injury and activate purinergic receptors expressed on the Schwann cell surface, but little is known about the involvement of purine signalling in wound healing. We studied the effect of UTP on Schwannoma cell migration and wound closure and the intracellular signaling pathways involved. We found that UTP treatment induced Schwannoma cell migration through activation of P2Y_2_ receptors and through the increase of extracellular matrix metalloproteinase-2 (MMP-2) activation and expression. Knockdown P2Y_2_ receptor or MMP-2 expression greatly reduced wound closure and MMP-2 activation induced by UTP. MMP-2 activation evoked by injury or UTP was also mediated by phosphorylation of all 3 major mitogen-activated protein kinases (MAPKs): JNK, ERK1/2, and p38. Inhibition of these MAPK pathways decreased both MMP-2 activation and cell migration. Interestingly, MAPK phosphorylation evoked by UTP exhibited a biphasic pattern, with an early transient phosphorylation 5 min after treatment, and a late and sustained phosphorylation that appeared at 6 h and lasted up to 24 h. Inhibition of MMP-2 activity selectively blocked the late, but not the transient, phase of MAPK activation. These results suggest that MMP-2 activation and late MAPK phosphorylation are part of a positive feedback mechanism to maintain the migratory phenotype for wound healing. In conclusion, our findings show that treatment with UTP stimulates *in vitro* Schwannoma cell migration and wound repair through a MMP-2-dependent mechanism via P2Y_2_ receptors and MAPK pathway activation.

## Introduction

Peripheral nerve injury initiates a sequence of events through which macrophages and Schwann cells clear damaged axons and potentiate axonal regeneration and nerve remyelination [1]. During Wallerian degeneration, de-differentiating Schwann cells secrete growth factors and neurite-promoting factors to guide the growing axon, as well as proteolytic enzymes to remodel extracellular matrix (ECM) and facilitate cell migration [2], [3], [4], [5]

One important class of proteases are matrix metalloproteinases (MMPs), a large family of zinc- and calcium-dependent enzymes that act on a wide number of extracellular and cell surface receptors, ligands, and adhesion and structural proteins [6], [7]. MMP upregulation has been linked to the pathogenesis of peripheral nerve damage [8], [9], [10], [11]. Specifically, MMP-2 and MMP-9 have been involved in remodeling of the ECM during nerve degeneration and regeneration [12], [13], [14], [15]. MMP-2 and MMP-9 are highly expressed after sciatic nerve injury: MMP-9 activity increases acutely at the site of injury some hours after nerve crush, whereas MMP-2 activity is delayed but maintained during nerve regeneration proximally and distally to the injury site, suggesting that MMP-2 acts to facilitate axonal extension along the nerve matrix [10]. In spinal cord injury, the same pattern is observed: MMP-9 activity is highly increased 12 to 24 hours after injury to facilitate leukocyte infiltration while MMP-2 increases its activity 5 to 14 days after injury to facilitate nerve recovery and limit the formation of a glial scar [16], [17], [18]. In Schwann cells, MMP-9 is required for insulin-like growth factor (IGF) release and the subsequent activation of the MEK/ERK pathway via IGF-1 and ErbB receptors [19]. MMP-9 also activates the Akt/ERK pathway and promotes migration by binding to the low-density lipoprotein receptor-related protein [20]. Taking into account these findings, the modulation of MMP activity may be a relevant target to enhance regeneration in demyelinating diseases of the peripheral nervous system (PNS) [17].

There is a growing body of evidence implicating purinergic P2Y receptors in cell communication, migration, and wound repair in response to injury in many cell types [21], [22], [23], [24]. After injury, nucleotides released from cells activate the purinergic receptor-signaling pathway to mediate the response to injury [25]. Nucleotide binding to P2Y receptors, which are coupled to the protein Gα_q_, activates phospholipase C_β_ (PLCβ). PLCβ cleaves phosphatidylinositol 4,5-biphosphate (PIP_2_) to diacylglycerol (DAG) and phosphoinositol tri-phosphate (IP_3_), resulting in the release of intracellular Ca^2+^ from endoplasmic reticulum stores. The increase in cytosolic Ca^2+^ induces a myriad of alterations in the tyrosine phosphorylation of proteins ranging from adhesion molecules to members of the mitogen- activated protein kinase (MAPK) family [26], [27]. MAPKs such as c-Jun N-terminal protein kinase (JNK), extracellular signal-regulating kinase (ERK), and p38 transduce extracellular signals into various cellular responses such as proliferation, differentiation, and migration [28], [29], [30], [31]. Accumulating evidence suggests that these MAPKs play a role in the migration of various cell types [32], [33], [34], [35].

Although the activation of P2Y purinergic receptors is known to activate a MAPK signaling cascade, the role of the purinergic signaling pathway in relation with Schwann cell migration and wound repair has not yet been described. The present study aimed to determine the effect of extracellular uridine 5′-triphosphate (UTP) on Schwannoma cell migration and wound repair and to establish whether MMP-2 is involved in this effect. For the first time, we report that UTP stimulates *in vitro* Schwannoma cell migration and wound repair through a MMP-2-dependent mechanism via P2Y_2_ receptors and MAPK pathway activation.

## Materials and Methods

### Reagents

Dulbecco's Modified Eagle's Medium (DMEM), penicillin, streptomycin, and glutamine were purchased from PAA (Linz, Austria). Donor bovine serum (DBS) was purchased from Gibco (Rockville, MD, USA). Suramin, PBS, Hoechst 33342, trypan blue, forskolin, pituitary extract, protease and phosphatase inhibitor cocktails, SB203580, SP600125, U0126, and UTP were purchased from Sigma–Aldrich (St Louis, MO, USA). GM6001 was purchased from Merck Millipore (Billerica, MA, USA). All other reagents used were of analytical grade.

### Schwann cell line cultures

The rat schwannoma cell line RT4-D6P2T was purchased from the European Collection of Cell Cultures (#93011415; ECACC, Salisbury, UK) and maintained in DMEM high glucose medium supplemented with 2 mM L-glutamine, 50 U/mL penicillin, 50 mg/L streptomycin, and 10% (v/v) DBS. Cultures were incubated in a 5% CO_2_ humidified atmosphere at 37°C. Cells were seeded at a density of 1.2×10^5^ cells/cm^2^ and starved in 1% (v/v) DBS for 24 h before nucleotide treatment.

### Schwann cell primary cultures

Schwann cells were isolated from the sciatic nerves of Sprague–Dawley rats on postnatal days 7 to 10 as previously described [36]. After chemical and mechanical dissociation, cells were cultured on dishes in DMEM high glucose medium supplemented with 10% (v/v) DBS, 5 µM forskolin, 20 µg/mL pituitary extract, 2 mM glutamine, 100 U/mL penicillin and 50 mg/L streptomycin. To eliminate fibroblasts, cells were replated on poly-l-lysine and laminin-coated culture dishes or wells according to the Kreider group [37]. In the resulting cultures, Schwann cells had a purity of more than 83% (Hoechst and S100 immunoreactivity). Cells were used for experiments at 7 to 10 days in culture.

### Sodium dodecyl sulfate polyacrylamide gel electrophoresis (SDS-PAGE) and immunoblotting

Schwann cells were washed with PBS (pH 7.4) and lysed in buffer that contained 50 mM Tris–HCl (pH 7.4), 150 mM NaCl, 1 mM EDTA, 1% (v/v) NP-40, and protease and phosphatase inhibitors. The lysates were incubated on ice for 10 min and sonicated. Protein concentrations were measured using the Bradford method. Thereafter, 30 µg of cell lysate was denatured with sample buffer [50 mM Tris–HCl, 2% (v/v) SDS, 100 mM DTT, 10% (v/v) glycerol, pH 6.8], subjected to 10% SDS-PAGE, and transferred onto Immobilon-P membranes (Merck Millipore). The membranes were blocked for 1 h with 5% (w/v) dried skimmed milk in TBS-T buffer [50 mM Tris, 150 mM NaCl, 100 mM KCl, and 0.1% (v/v) Tween-20, pH 7.4], and incubated with the following primary antibodies: rabbit anti-phospho-ERK1/2 (9101; Cell Signaling Technology, Beverly, MA, USA; 1∶1000), rabbit anti-ERK1/2 (9102; Cell Signaling Technology; 1∶1000), rabbit anti-phospho-JNK (9251; Cell Signaling Technology; 1∶1000), rabbit anti-JNK (9252; Cell Signaling Technology; 1∶1000), rabbit anti-phospho-P38 (ab4822; Abcam; 1∶1000), rabbit anti-P38 (ab27986; Abcam; 1∶1000), rabbit anti-P2Y_2_ (ab10270; Abcam; 1∶1000), rabbit anti-MMP-2 (ab37150; Abcam; 1∶500), and mouse anti-GAPDH (AM4300; Applied Biosystems, Carlsbad, CA, USA; 1∶20000). Antibody binding was detected with the corresponding HRP-coupled secondary antibody (Calbiochem; 1∶5000), and the bands were visualised using the Luminata Forte detection system (Merck Millipore). The immunoreactive signals were quantified using Image J software (National Institutes of Health, USA).

### Gelatin zymography

Conditioned culture medium was collected and centrifuged at 15,000 rpm for 5 min at 4°C. The cleared medium was concentrated 10-fold using Centricon with a 10-kDa pore size (Merck Millipore). Samples (20 µg protein) of Schwann cell-conditioned media were then resolved on 10% SDS-PAGE gels containing 0.1% (w/v) gelatin as the protease substrate. After electrophoresis, gels were washed with 100 mM Tris-HCl (pH 7.5) and 2.5% (v/v) Triton X-100 for 30 min and subsequently incubated for 16 h at 37°C in digestion buffer containing 100 mM Tris-HCl (pH 7.5), 5 mM CaCl_2_, and 200 mM NaCl. Bands corresponding to activity were visualized by negative staining using 0.25% (w/v) Coomassie Brilliant Blue R-250 (Bio-Rad, CA, USA) for 6 hours followed by destaining for 30 min in a solution of 10% (v/v) acetic acid and 25% (v/v) isopropanol. To quantify the MMP-2 activity, the intensities of negative stained bands were analyzed using Image J software (National Institutes of Health, USA). MMP-2 activity was expressed as the active MMP-2 intensity per total MMP-2 intensity (pro-MMP-2 plus active MMP-2).

### Immunocytochemistry

Schwann cells cultured on coverslips were fixed with 4% (v/v) paraformaldehyde and 4% (w/v) sucrose in PBS, permeabilized by incubation for 20 min with methanol in a solution that comprised PBS–Triton (0.2%; v/v), 0.5% (w/v) BSA, and 20 mM glycine, and then labelled with Hoechst 33342 (1 µg/mL) for 1 h at 25°C in the dark. For MMP-2 staining, coverslips were incubated for 4 h with mouse anti-MMP-2 (ab37150; Abcam; 1∶500) and fluorescent secondary antibody conjugated with Alexa Fluor (Invitrogen, 1∶2000) for 1 h. Finally, coverslips were mounted with the anti-fading medium Fluoromount (Sigma) and fluorescence microscopic images were obtained using a laser confocal microscope (Leica DM IRB, Wetzlar, Germany). The immunofluorescence intensity was quantified using MetaMorph® Software (Molecular Devices, LLC).

### Gelatinase activity

Gelatinase activity was determined with *in situ* zymography using DQ-Gelatin fluorescein conjugate as a substrate. Schwannoma cells cultured on coverslips were washed with 0.5 M Tris-HCl (pH 7.6), 1.5 M NaCl, 50 mM CaCl_2_, and 2 mM NaN_3_. Cells were then treated with DQ-Gelatin (100 µg/mL) at 37°C for 2 h (EnzCheck Gelatinase Assay, Molecular Probes, USA). Finally, coverslips were mounted with the anti-fading medium Fluoromount (Sigma) and fluorescence microscopy images were obtained using a laser confocal microscope (Leica DM IRB). The number of fluorescence spots in each cell was counted in different regions and the results were expressed as number of positive MMP-2 Schwann cells per area.

### Wound -healing assay

Schwann cells were seeded at 2.5×10^5^ cells/cm^2^ and incubated for 48 h to allow for the formation of a monolayer. A wound was created in the monolayer with a 1000 µL pipette tip. The cells were then washed with PBS (pH 7.4) and replaced with DMEM high glucose medium supplemented with 2 mM L-glutamine, 50 U/mL penicillin, 50 mg/L streptomycin, and 1% (v/v) DBS. Cultures were incubated in a 5% CO_2_ humidified atmosphere at 37°C. Images of 4 random fields along the wound were taken at different times using a Nikon camera connected to a Nikon inverted light microscope. Wound closure was quantified by measuring the percentage of the occupied wound area at different times using the TScratch v1.0 program (Matlab, Zurich, Switzerland). The rate of migration (velocity) was expressed as occupancy (%) per hour.

### Migration assay

Schwann cell migration assays were performed using BD BioCoat cell culture inserts with 6.5-mm-diameter filters with an 8-µm pore size according to the manufacturer's instructions (BD Biosciences). Schwann cells at a density of 1.5×10^5^ cells/insert were seeded into the upper chamber in complete medium: DMEM high glucose medium supplemented with 2 mM L-glutamine, 50 U/mL penicillin, 50 mg/L streptomycin, and 1% (v/v) DBS. Complete medium with UTP was placed in the lower chamber as the chemoattractant. After incubation in a 5% CO_2_ humidified atmosphere at 37°C, the migrated cells in the lower chamber were fixed with 70% (v/v) ethanol and stained with 0.2% (w/v) Crystal Violet. For each membrane, the number of cells was counted in 4 blindly chosen random fields under light microscopy. The fold increase in cell migration was expressed by taking the control value as 1.

### Quantitative reverse transcription polymerase chain reaction (RT-PCR)

The total RNA from 10^6^ Schwann cells was isolated using TRIzol reagent (Invitrogen, Paisley, UK) and reverse transcribed using an iScript cDNA synthesis kit (Bio-Rad, Berkeley, CA, USA). The cDNA template underwent quantitative analysis of cDNA amplification, which was assessed using SYBR Green (Bio-Rad). Standard PCRs were performed with the following specific primers (100 mM each): MMP-2 (NM_031054), forward, 5′-AGG GAA TGA GTA CTG GGT CT-3′, reverse, 5′-CAG TTA AAG GCA GCG TCTA C-3′; P2Y_2_ receptor (NM_017255.1), forward, 5′-CTG CCA GGC ACC CGT GCT CTA CTT-3′, reverse, 5′-CTG AGG TCA AGT GAT CGG AAG GAG-3′; P2Y_4_ receptor (NM_031680.1), forward, 5′-CAC CGA TAC CTG GGT ATC TGC CAC-3′, reverse, 5′-CAG ACA GCA AAG ACA GTC AGC ACC-3′; P2Y_6_ receptor (NM_57124.1), forward, 5′-GGA GAC CTT GCC TGC CGC CTG GTA-3′, reverse, 5′-TAC CAC GAC AGC CAT ACG GGC CGC-3′; glyceraldehyde-3-phosphate dehydrogenase, GAPDH (NM_017008.3), forward, 5′-TGG GAA GCT GGT CAT CAA C-3′, reverse, 5′-GCA TCA CCC CAT TTG ATG TT-3′. The NM number indicates the accession number of each gene in the NCBI nucleotide database. All amplified PCR products were verified by melting curve analysis. Quantification was performed by the ΔΔC_T_ method, and levels of mRNA expression were normalised to the housekeeping gene that encodes GAPDH.

### Short hairpin RNA

Knockdown Schwannoma cells were generated using short hairpin RNA directed against the rat MMP-2 gene (shMMP-2) constructed in the pLVTHM-GFP plasmid vector (Addgene, Cambridge, USA). Sequence targeting for MMP-2 was selected based on the rules for RNA susceptibility proposed by Tuschl's group [38] and using the siRNA prediction program from the Whitehead Institute for Biomedical Research [39]. Two complementary DNA oligonucleotides (Roche, Berlin, Germany) were annealed to produce a double-stranded DNA fragment encoding a 19-nucleotide sense strand, a 9-nucleotide loop, and a 19-nucleotide antisense strand that correspond to bases 2173–2191 in MMP-2 mRNA (NM_031054). The sequence of MMP-2 shRNA is as follows: sense, 5′-CGC GTC CCC GGA GAT ACA ATG AAG TAA ATT AAG AGA TTT ACT TCA TTG TAT CTC CTT TTT GGA AAT-3′ and antisense, 5′-CGA TTT CCA AAA AGG AGA TAC AAT GAA GTA AAT CTC TTG AAT TTA CTT CAT TGT ACT CCG GGG A-3′. A plasmid carrying a random sequence was used as a shRNA control (shRandom). The sequence of the shRNA-Random is as follows: sense, 5′-GAT CCC CGC AGT GCA ATA TCG GAA ACT TCA AGA GAG TTT CCG ATA TTG CAC TGC TTT TT-3′ and antisense, 5′-AGC TAA AAA GCA GTG CAA TAT CGG AAA CTC TCT TGA AGT TTC CGA TAT TGC ACT GCG GG-3′. Duplex DNAs of shRNA-MMP-2 and shRNA-Random were cloned into the *ClaI* and *MluI* sites of the pLVTHM vector. The shRNA against P2Y_2_ was a generous gift from Dr. Díaz-Hernández and Dr. Miras-Portugal (Universidad Complutense de Madrid, Spain). RT4-D6P2T cells were transfected with different shRNAs using Lipofectamine 2000 in accordance with the manufacturer's instructions (Invitrogen). Efficacy of shMMP-2 and shP2Y_2_ was tested in transiently transfected cells using immunoblotting for the protein and quantitative RT-PCR for mRNA expression (see Supporting Information, Fig. S1).

### Statistical Analysis

Results were expressed as the mean ±SD of results from at least 3 independent experiments. Statistical analyses were carried out using the GraphPad Prism 5.0 Software package (ANOVA analysis plus Newman–Keuls post-test: *P≤0.05, **P≤0.01, and ***P≤0.001).

### Ethics Statement

Animal experimental procedures were submitted and authorized by the Ethics Committee of the Universitat Internacional de Catalunya (CEREA-UIC 2009).

## Results

### UTP enhances Schwann cell migration and wound repair

**Figure 1:**
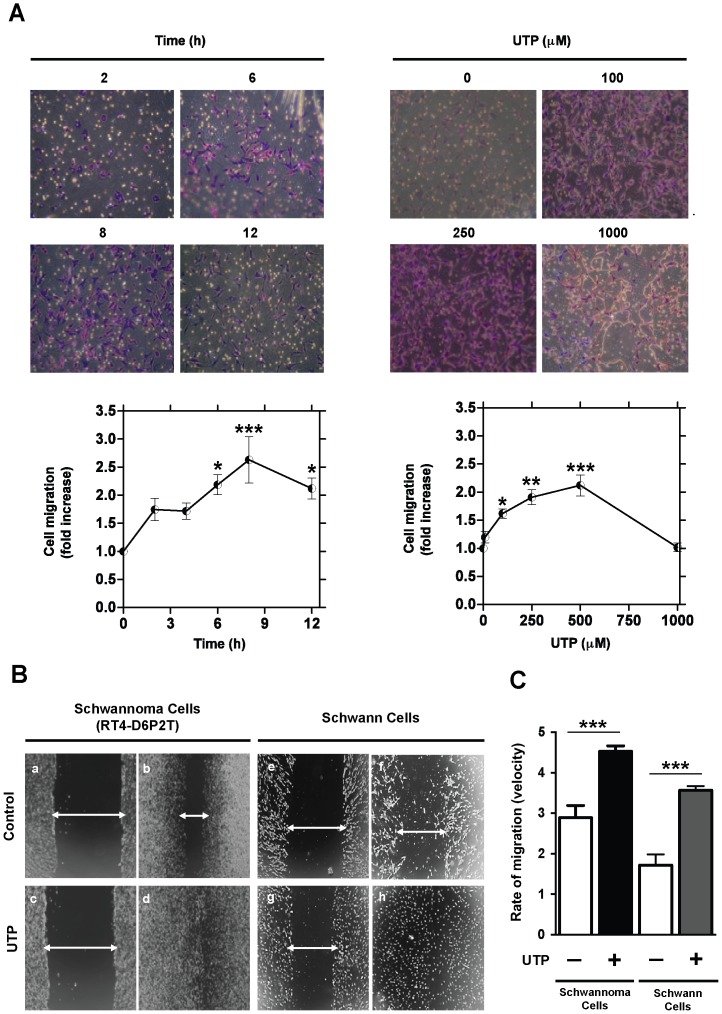
UTP enhances Schwann cell migration and wound repair. (**A**) Transwell migration assay for RT4-D6P2T cells. Cells were seeded in the upper side of a transwell membrane and treated with UTP (250 µM) at different times (2, 4, 6, 8, 10, and 12 h), or incubated for 12 h with various UTP concentrations (0, 10, 100, 250, 500, and 1000 µM). Representative images (objective magnification ×10) of the dose–response and time–course transwell migration studies are shown. Migrated cells were stained with crystal violet and counted. Data were expressed as the fold increases of cell migration when compared to untreated cells. Values were calculated as the mean ± SD using 3 independent experiments. Statistical significance: *P≤0.05, **P≤0.01, and *** *P*≤0.001. (**B**) Wound-healing assay for Schwann cell line and for primary Schwann cells. Monolayer cells for both Schwann cultures were scraped (a, c, e, and g) and either untreated (b and f) or treated with 250 µM UTP (d and h). Micrographs are representative of at least 3 independent experiments (objective magnification ×10). (**C**) Quantitative analysis of the rate of migration (velocity) calculated as percentage of wound occupancy per hour. Values were calculated as the mean ± SD of 3 independent experiments. Statistical significance: *** *P*≤0.001.

In response to injury, Schwann cells increase their migration to cover the damaged area. Previous studies on microglial, smooth muscle, and epithelial cells have demonstrated that nucleotides trigger wound repair [22], [24], [40]. To investigate the ability of the nucleotide UTP to increase Schwannoma RT4-D6P2T cell migration, dose–response and time–course studies were performed using the Transwell migration assay (Fig. 1A). Dose–response studies revealed that cell migration significantly increased after UTP treatment (12 h, 100–500 µM). As previously reported, the observed decrease in cell migration at 1 mM is due to UTP cytotoxicity [41]. As extracellular concentration of UTP is greatly enhanced after injury [42], [43], a high UTP concentration (250 µM) was used in all the experiments. On the other hand, time–course analysis showed that cell migration significantly increased between 6 and 12 h after UTP treatment (250 µM).

The effect of UTP on wound repair was also confirmed in Schwann cell line and in primary cultured Schwann cells. Treatment of these cells with UTP (250 µM) significantly reduced the time required to close the wound completely (100%). RT4-D6P2T cells incubated with UTP filled the wound after 20 hours (Fig. 1B, d) compared to control cells, which occupied only 56±8% of the wound area in that time (Fig. 1B, b). Likewise, primary cultured Schwann cells treated with UTP filled the wound after 36 hours (Fig. 1B, h), while untreated cells occupied only 57±12% of the wound area in that time (Fig 1A, f). Quantitative analysis of the wound–healing assay represented in Figure 1C revealed that UTP treatment increases Schwann cell velocity 2.1-fold (from 1.7±0.5% to 3.6±0.2% of occupancy/h) for primary Schwann cells and a 1.6-fold increase (from 2.9±0.5% to 4.5±0.2% of occupancy/h) for the RT4-D6P2T cell line.

### MMP-2 activation by UTP is essential for Schwann cell wound repair

**Figure 2:**
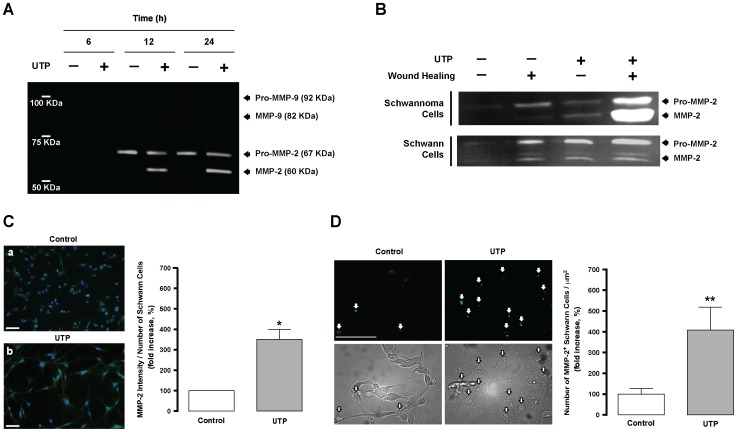
UTP regulates MMP-2 activity and expression. (**A**) Time–course gelatin zymography analysis for Schwann cell line. Conditioned media from 2.5×10^6^ RT4-D6P2T cells that were either treated or untreated with UTP (250 µM) at different times (6, 12, and 24 hours) were loaded on SDS-PAGE gel containing gelatin. The zymogram is representative of 3 independent experiments. (**B**) Gelatin zymograms for a Schwann cell line and primary Schwann cells after UTP and wound healing treatments. Conditioned media from Schwann cells were collected after 12 h of UTP (250 µM) or wound healing treatments. Equal amounts of protein (20 µg) were loaded on SDS-PAGE gel containing gelatin. (**C**) Representative dual-fluorescence labeling of MMP-2 (green) and nuclei (blue) using specific antibody against MMP-2 and Hoechst staining and quantitative analysis for primary Schwann cells (a and b, objective magnification ×40). Scale bar: 50 µm. Statistical significance: *P≤0.05 compared to control cells. (**D**) In situ zymography images and quantitative analysis of gelatinase activity in RT4-D6P2T cells. Scale bar: 50 µm. Statistical significance: **P≤0.01 compared to control cells.

MMPs in their active form have the potential to promote cell migration and are involved in wound repair [16]. In order to determine whether UTP is able to induce MMP activation, the activity of MMP-2 and MMP-9 in the conditioned media was studied using gelatin zymography in RT4-D6P2T cells after UTP treatment (250 µM). A time–response study revealed that MMP-2 becomes active (proteolytically cleaved) after 12 h to 24 h of UTP treatment, whereas MMP-9 was not detected at any time during the study (Fig. 2A). In the case of MMP-2, the pro-form of the protein (67 kDa) showed similar levels in both controls and UTP-conditioned media after 12 h to 24 h of incubation. By contrast, the activity of the cleaved form of MMP-2 (60 kDa) appeared to be clearly enhanced in UTP-conditioned medium (Fig. 2A). After assessing UTP-induced MMP-2 activation in RT4-D6P2T cells, we investigated whether MMP-2 activation was induced by wound. Interestingly, gelatin zymography experiments showed that MMP-2 was activated during wound healing (Fig. 2B), with increased activation in the presence of UTP (250 µM, 12 h). This amplified signal of the active form of MMP-2 was observed in the Schwann cell line as well as the primary Schwann cell culture (Fig. 2B). These results suggest that either the UTP nucleotide or the conditioned medium from wounded cultures induce the activation of MMP-2. The mRNA levels in RT4-D6P2T and primary Schwann cells were also evaluated by quantitative RT-PCR. Results showed that UTP significantly increases MMP-2 expression in both cell models after 6-24 hours of treatment (Fig. S2).

Immunocytochemistry studies corroborated that MMP-2 protein expression is increased in primary Schwann cells (Fig. 2C, b) treated with UTP (250 µM, 24 h) compared to control cells (Fig. 2C, a). Quantitative analysis showed that the MMP-2 immunofluorescence intensity was significantly increased (p = 0.018) in UTP-treated cells compared to untreated cells (Fig. 2C). Furthermore, endpoint analysis of fluorescence-quenched gelatin degradation in RT4-D6P2T cells revealed a significant increase (p = 0.002) in the number of MMP-2 positive cells in UTP-treated cells compared to untreated control cells (Figure 2D). These findings are in agreement with the results obtained by gelatin zymography and gene expression analysis and corroborate that UTP enhances MMP-2 activation and expression.

**Figure 3:**
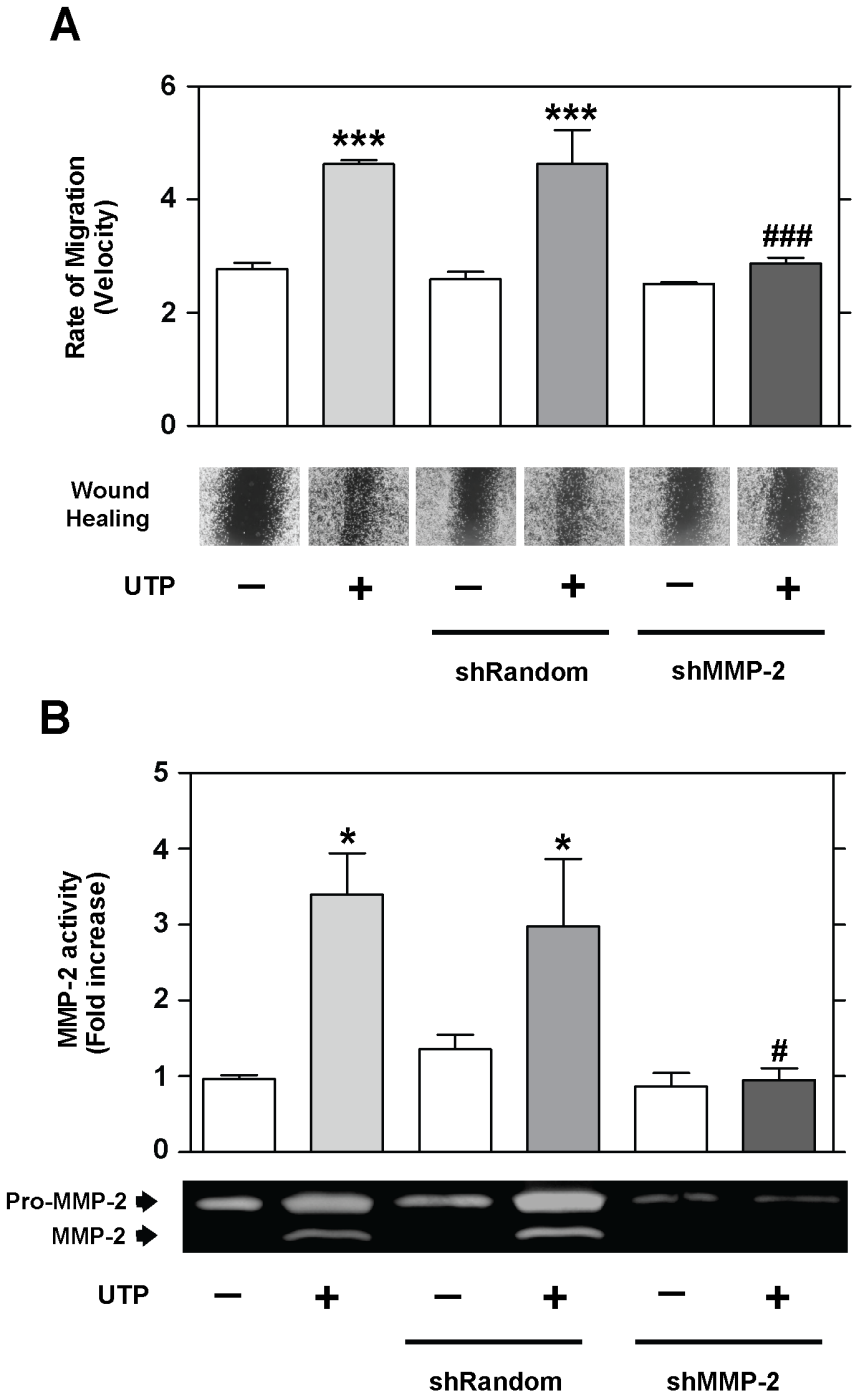
UTP regulates MMP-2 activity. Wound healing and gelatin zymograms of RT4-D6P2T cells transfected with shRNA directed against the MMP-2 gene (shMMP-2) and control cells (non-transfected cells or cells transfected with shRandom sequence). Representative images (objective magnification ×10) of wound healing and gelatin zymograms and quantitative analysis of the rate of migration (velocity) and MMP-2 activity are shown. Values were calculated as the mean ± SD using 3 independent experiments. Statistical significance: *P≤0.05 and ***P≤0.001 when compared to control cells; #*P*≤0.05 and ###*P*≤0.001 when compared to UTP-treated cells.

Finally, to evaluate the role of activated MMP-2 in Schwannoma cell wound repair, a short hairpin RNA (shRNA) directed against the rat MMP-2 gene (shMMP-2) was developed. Following shMMP-2 transfection, substantial knockdown of the MMP-2 expression was observed in RT4-D6P2T cells at both mRNA (81.3±0.2% decrease) and protein (52.3±0.7% decrease) levels compared with transfected (shRandom) and nontransfected control cells (see Supporting Information, Fig. S1). Wound healing and zymography assays showed a significant decrease in the rate of migration (*P*≤0.001; Fig. 3A) and MMP-2 activation (*P*≤0.05; Fig. 3B) in MMP-2-silenced RT4-D6P2T cells when compared to control cells. Altogether, these results strongly suggest that UTP-activated MMP-2 plays an important role in Schwann cell wound repair.

### P2Y_2_ receptor is involved in Schwannoma cell wound repair and MMP-2 activation

**Figure 4:**
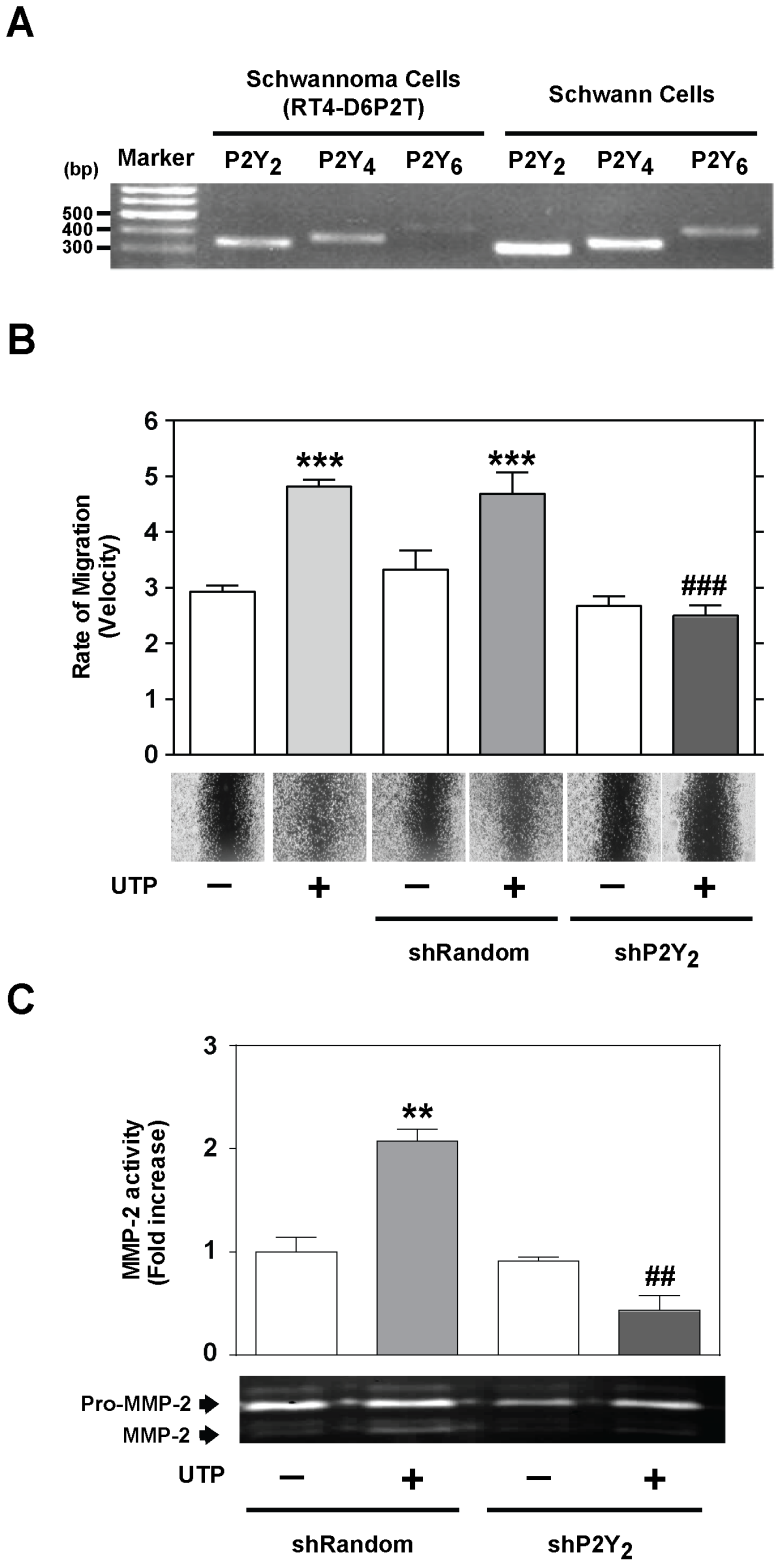
P2Y_2_ receptors are necessary for Schwann cell line wound repair. (**A**) RT-PCR amplification with specific primers against P2Y_2_, P2Y_4_, and P2Y_6_ receptor subtypes was performed in RT4-D6P2T cells and in primary Schwann cells after the isolation of total RNA. PCR products were separated on a 1% agarose gel and visualized with ethidium bromide. (**B-C**) Wound healing and gelatin zymograms of RT4-D6P2T cells transfected with shRNA directed against the P2Y_2_ gene (shP2Y_2_) and control cells (non-transfected cells or cells transfected with shRandom sequence). Representative images (objective magnification ×10) of wound healing and gelatin zymograms and quantitative analysis of the rate of migration (velocity) and MMP-2 activity are shown. Values were calculated as the mean ± SD using 3 independent experiments. Statistical significance: **P≤0.01 and ***P≤0.001 when compared to control cells; ##*P*≤0.01 and ###*P*≤0.001 when compared to UTP-treated cells.

The expression of the uridine-sensitive P2Y receptors (P2Y_2_, P2Y_4_ and P2Y_6_) was determined by RT-PCR in RT4-D6P2T cells and in primary Schwann cell culture. As shown in Figure 4A, three DNA fragments of 339, 377, and 450 bp were amplified. These fragments matched the predicted sizes of the amplifications products for the P2Y_2_, P2Y_4_ and P2Y_6_ receptor subtypes, respectively. Given that P2Y_2_ receptor has been involved in cell migration of several cell types, their implication in Schwann cell wound repair was studied by using a shRNA directed against the P2Y_2_ gene (shP2Y_2_). A significant decrease in the rate of migration (*P*≤0.001; Fig. 4B) and MMP-2 activity (*P*≤0.01; Fig. 4C) in P2Y_2_-silenced RT4-D6P2T cells was observed by wound healing and zymography assays. This data suggest the involvement of P2Y_2_ receptor in the UTP-induced Schwann cell migration and MMP-2 activation.

**Figure 5:**
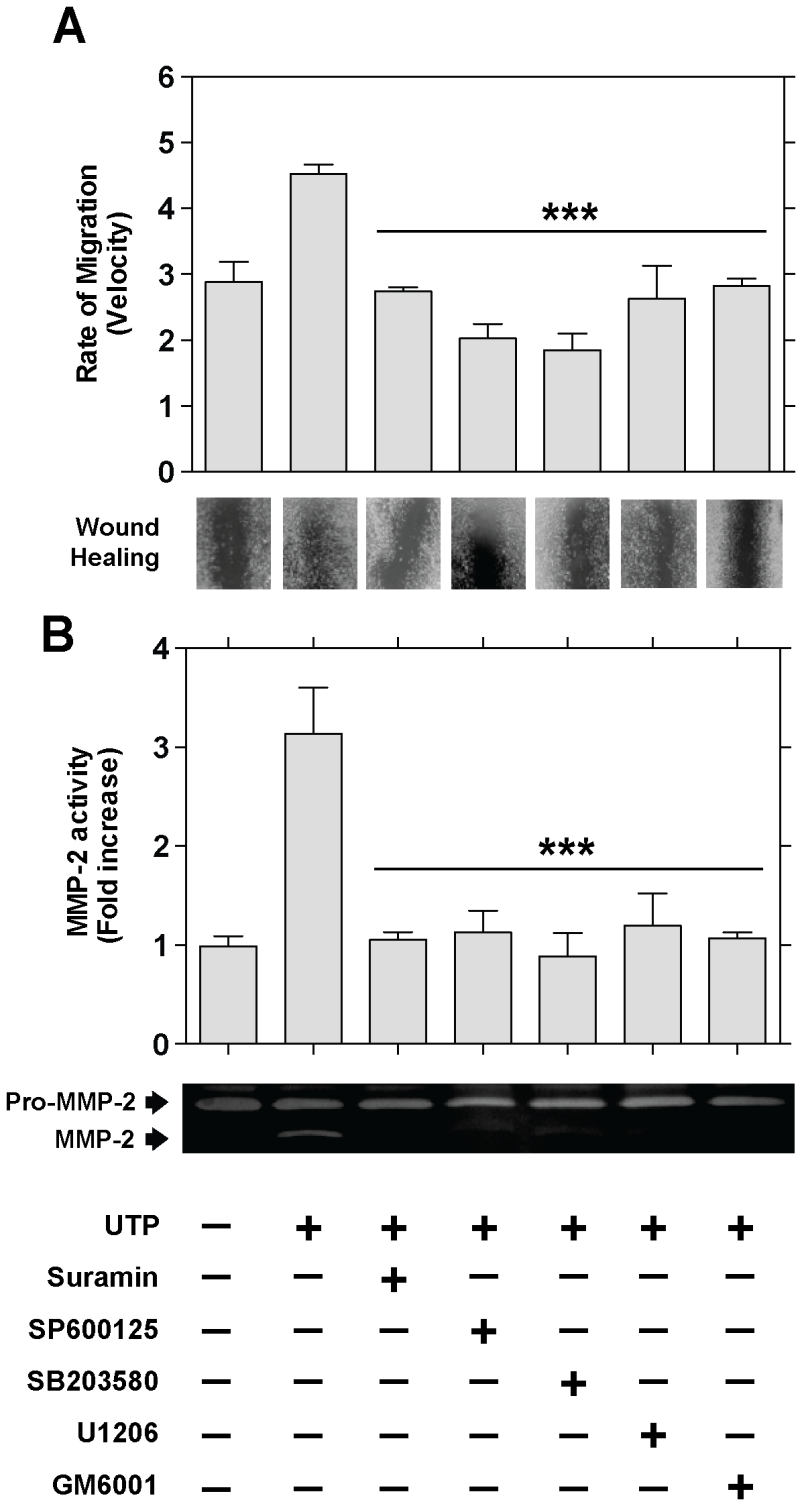
P2Y_2_ receptors are necessary for Schwann cell line wound repair. RT4-D6P2T cells were preincubated (30 min) with selective inhibitors of different proteins involved in the P2Y receptor signaling pathway: suramin (100 µM; P2Y_2_ receptor antagonist), SP600125 (20 µM; JNK inhibitor), SB203580 (10 µM; P38 inhibitor), U0126 (10 µM; ERK inhibitor), and GM6001 (10 µM; MMP inhibitor). After UTP treatment (12 h, 250 µM), the rate of migration and MMP-2 activity were determined. Representative images are shown below the corresponding graphs. Each bar represents the mean ± SD using 3 independent experiments. Statistical significance: ***P≤0.001 when compared to UTP-treated cells.

To investigate whether the signaling pathway through P2Y receptors is implicated in Schwannoma cell migration and MMP-2 activation, and given that P2Y receptors have been reported to induce MAPK activity [25], [44], we determined whether the P2Y-MAPK pathway mediated UTP-induced migration in Schwann cell line. RT4-D6P2T cells were pre-incubated for 20 min before UTP (250 µM) stimulation with various selective inhibitors: a) suramin, an antagonist at P2Y_1, 2, 3, 6, 11_ receptors [45], [46]; b) U0126, an inhibitor of MEK1/2 (upstream ERK1/2 kinase); c) SB203580, an inhibitor of P38; d) SP600125, an inhibitor of JNK; and e) the broad-spectrum MMP inhibitor GM6001. Results revealed a significant decrease (*P*≤0.001) in the rate of cell migration (Figure 5A) and MMP-2 activation (Figure 5B) after UTP treatment when cells were pre-incubated with 1 of the inhibitors, in comparison with UTP-treated cells.

### UTP induces biphasic MAPK phosphorylation

**Figure 6:**
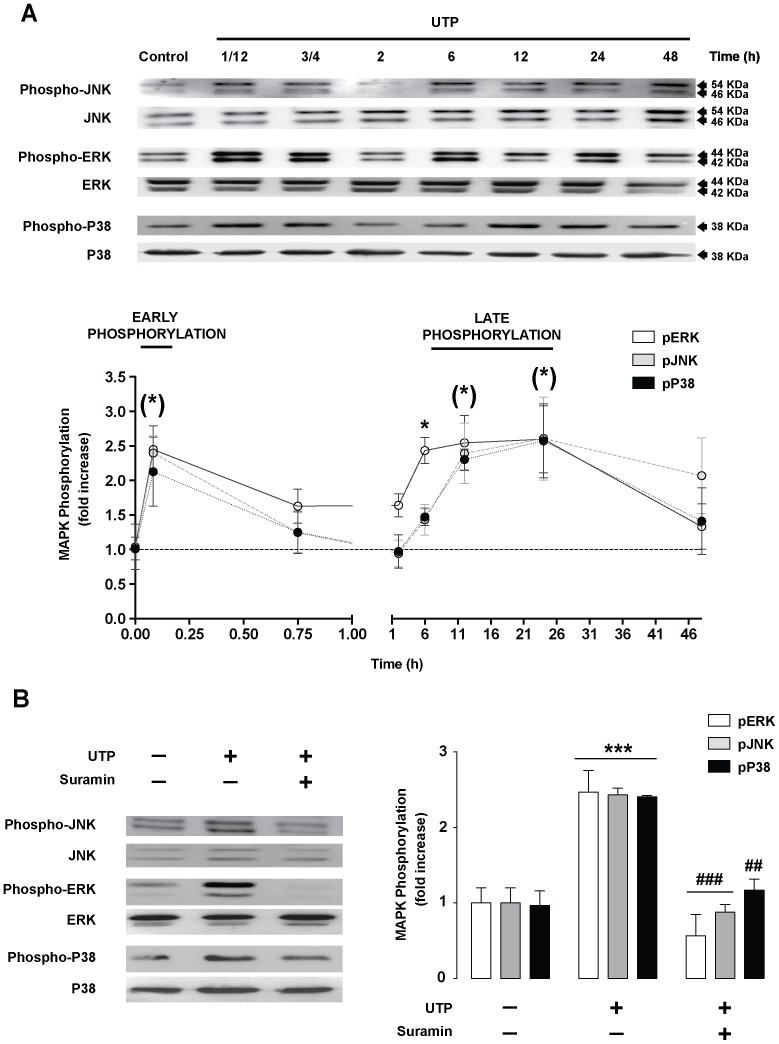
UTP induces biphasic MAPK phosphorylation. (**A**) Western blot analysis of time–course MAPK phosphorylation induced by UTP. RT4-D6P2T cells were incubated with UTP (250 µM) at the indicated times and equal amounts of protein (30 µg) were resolved in SDS-PAGE. Western blots were performed using antibodies against phosphorylated and total MAPK (ERK1/2, JNK, and P38). The ratio of phosphorylated MAPK to total MAPK was calculated by densitometry in each sample, and a value of 1 was given to the control cells. Representative western blots for each kinase are shown above the graphs. Blots are representative of 3 independent experiments. Statistical significance: *P≤0.05 compared to control cells. (B) RT4-D6P2T cells were preincubated (30 min) with suramin (100 µM; P2Y_2_ receptor antagonist). After UTP treatment (12 h, 250 µM), the ratio of phosphorylated MAPK to total MAPK was calculated by densitometry in each sample. Representative images are shown below the corresponding graphs. Each bar represents the mean ± SD using 3 independent experiments. Statistical significance: ***P≤0.001 when compared to control cells; ##*P*≤0.005, and ###*P*≤0.001 when compared to UTP-treated cells.

To further study UTP stimulation of MAPKs, time–course phosphorylation was investigated. RT4-D6P2T cells were treated with UTP and cell lysates were collected after various time intervals. Equal amounts of protein were resolved in SDS-PAGE and blotted with MAPK antibodies against phospho-JNK, phospho-ERK1/2, and phospho-P38. The same membranes were also blotted with antibodies against total (active and non-active) forms of MAPKs (JNK, ERK1/2 and P38). Stimulation of RT4-D6P2T cells with UTP (250 µM) resulted in a transient increase in all phosphorylated MAPK levels at 5 min (Fig. 6A: early phosphorylation), which decreased to near baseline levels at approximately 45 to 120 min after stimulation. A second wave of increased phosphorylated MAPK levels appeared at approximately 6 to 12 h and was sustained for up to 24 h (Fig. 6A: late phosphorylation). It was previously described that suramin was able to block the early UTP-induced phosphorylation [47]. Our data show that suramin pretreatment also blocked the late UTP-induced late phosphorylation (Fig 6B). These results suggest that UTP activates P2Y_2_ receptors, initiating downstream signaling cascades and resulting in biphasic MAPK phosphorylation.

### MMP-2 activation mediates late MAPK phosphorylation

**Figure 7:**
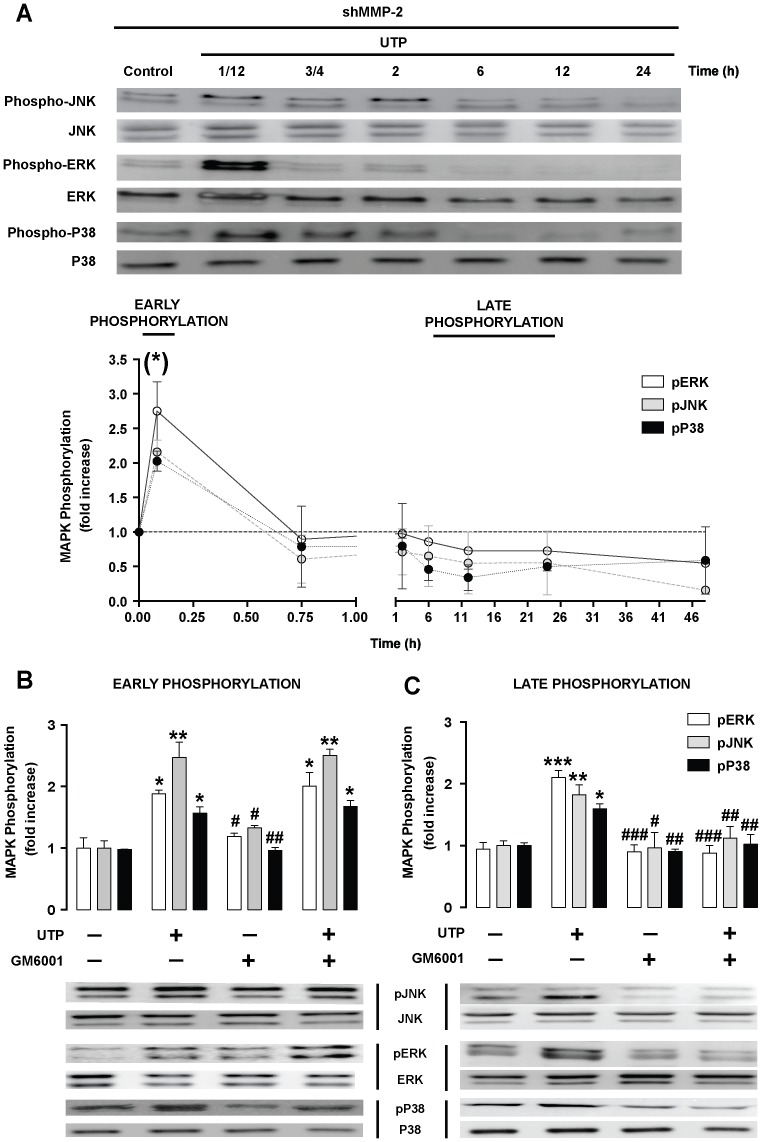
MMP-2 activation mediates late MAPK phosphorylation. (**A**) Western blot analysis of time–course MAPK phosphorylation induced by UTP. RT4-D6P2T cells transfected with shRNA against MMP-2 were incubated with UTP (250 µM) at the indicated times. (**B-C**) Schwannoma cells were preincubated for 30 min with a broad-spectrum MMP inhibitor (GM6001, 10 µM) and then incubated with UTP (250 µM) at either 5 min (early phosphorylation) or 12 hours (late phosphorylation). Proteins from cell lysates (30 µg) were resolved by SDS-PAGE and blotted against either phosphorylated or total MAPKs. Representative western blots for each kinase are shown above the graphs. Blots are representative of 3 independent experiments. Statistical significance: *P≤0.05, **P≤0.005, and ***P≤0.001 when compared to control cells; #*P*≤0.05, ##*P*≤0.005, and ###*P*≤0.001 when compared to UTP-treated cells.

To determine a possible relationship between MAPK phosphorylation and MMP-2 activation, MMP-2-silenced RT4-D6P2T cells were treated with UTP (250 µM) and cell lysates were collected at different times. Next, western blotting for 3 phosphorylated MAPKs was performed. Results showed that while early phosphorylation is similar to that in control cells, late MAPK phosphorylation is significantly downregulated in MMP-2-silenced cells (Fig. 7A). To confirm the involvement of active MMP-2 in late MAPK phosphorylation, RT4-D6P2T cells were stimulated with UTP in the absence or presence of a broad-spectrum MMP inhibitor (10 µM, GM6001) and MAPK phosphorylation levels were measured in cell extracts collected 5 min (early phosphorylation; Fig. 7B) or 12 h (late phosphorylation; Fig. 7C) after UTP treatment. GM6001 treatment did not inhibit the early increase but significantly (*P*≤0.05) reduced the UTP-stimulated late increase in phosphorylated MAPK levels. These results suggest that a UTP-induced late increase in MAPK phosphorylation is MMP-2-dependent.

## Discussion

Growing evidence suggests that nucleotides that are released upon injury stimulate nucleotide P2 receptors and serve as endogenous signals to induce a rapid wound healing response in glial cells [48], [49], [50]. In analogy with other G-protein-coupled receptors (GPCR), a cross-talk between P2Y and growth factor receptors may occur at different levels of the signal transduction pathway depending on receptor subtypes and the cell type and their respective environments [51], [52]. Extracellular nucleotides are known to function as chemotactic agents for microglial cells, smooth muscle cells, and epithelial cells [22], [24], [40], [53]. However, these studies did not explain the implication of the nucleotide UTP, a selective P2Y agonist, in injured Schwann cells. We hypothesize that UTP secreted to the medium during nerve injury binds P2Y receptors in the neighboring Schwann cells to induce MMP-2 activity and subsequent cell migration and wound recovery.

In the PNS, MMP-2 activity has been shown to increase after nerve injury and is associated with cell migration, axonal outgrowth, nerve regeneration, and remyelination [14], [17], [54]. Our results demonstrate that cell migration and wound repair significantly increased after UTP treatment in Schwann cells, and that the velocity of these processes depends directly on MMP-2 activation. We have also described that UTP significantly increases MMP-2 gene expression and that knockdown of MMP-2 is enough to completely suppress UTP-induced cell migration and MMP-2 activation in the extracellular media.

Intracellular calcium measurements clearly indicate that UTP-recognizing P2Y_2_ and P2Y_4_ receptors are functional in Schwann cells [41]. Given that UTP is hydrolyzed by ectonucleotidases to UDP, which is an agonist at P2Y_6_ receptor, we first identified the P2Y receptor subtype that mediates Schwann cell migration and MMP-2 activation. To that end, we used a pharmacological approach (P2Y_2_ is suramin-sensitive) and P2Y_2_-knowdown of Schwannoma cells, which suggested a predominant role of P2Y_2_ receptors in this migratory effect. Although UTP and ATP are equivalent in stimulating P2Y_2_ receptor, we decided to study the effect of UTP in order to avoid possible cross-signalling pathway induced by the activation of P2X receptors by ATP [55]. Then, our findings are consistent with previous reports demonstrating that P2Y_2_ receptors are involved in cell migration in several cell types, providing further evidence of the functional role of P2Y_2_ receptor during injury [56], [57], [58], [59], [60]. Indeed, P2Y_2_ receptors have been shown to be upregulated in a variety of tissues in response to injury [61]. All together, our data support the hypothesis that activation of P2Y_2_ receptors by extracellular UTP might regulate a reparative mechanism in response to wound injury.

Our previous work, and that of other authors, showed that P2Y receptor activation by UTP increases the expression of N-cadherin, an adhesion molecule related to Schwann cell migration, and that this increase is mediated by MAPK activation [47], [62]. In the present study, we also demonstrate that UTP-induced MMP-2 activation is dependent on MAPK signaling pathways. Indeed, after nerve injury, increased activity in multiple pathways—including the ERK/MAPK, JNK/c-Jun, Notch, and JAK/STAT (Janus kinase/signal transducer and activator of transcription) pathways—can be detected in Schwann cells [63], [64]. Furthermore, some authors suggest that ERK and JNK activity defines the state of Schwann cell differentiation. Whereas basal levels are necessary for the differentiation of precursors, elevated ERK and JNK activities drive dedifferentiation and migration [65], [66], [67], [68]. By contrast, the role of p38 MAPK in Schwann cells is controversial; it has been associated with the regulation of myelination [69], but also with a negative regulatory role in differentiation and myelination [70].

As previously described in many growth factors and cytokines, UTP treatment also results in a biphasic phosphorylation of MAPKs [71], [72]. This kinetic profile has recently been proposed as a model to allow a common signaling system to play distinct regulatory roles depending on the temporal duration of activation [73]. Although P2Y activation by UTP is well known to be involved in early MAPK phosphorylation, the role of UTP in late MAPK phosphorylation has not been described until now. Our data suggests that the initial transient phase of MAPK activation in Schwannoma cells is mediated by P2Y receptors [25], [47], whereas the late sustained phosphorylation is also mediated by MMP-2 activation. One possible explanation for late MAPK phosphorylation is that active MMP-2 in the extracellular space cleaves a membrane-anchored growth factor on Schwannoma cells. Many proteins released by proteolysis of the ECM proteins are known to control cell response and may act as effectors for Schwann cell migration [74]. Multiple lines of evidence indicate that growth factors, such as neuregulin-1 (NRG1), are involved in Schwann cell migration and myelination [75]. Furthermore, NRG1 expression and its putative receptors, ErbB2 and ErbB3, are known to be induced in injured sciatic nerves in rats, promoting Schwann cell proliferation and dedifferentiation in injured nerves [76], [77]. Thus, the cleavage of a growth factor (NRG1 type III) by MMP-2 and the subsequent phosphorylation of the ErbB transmembrane tyrosine kinase receptor could explain the late MAPK phosphorylation observed in Schwannoma cells after UTP treatment. This proposed hypothesis is based on previous observations demonstrating that MMP-9 regulates MAPK pathways through IGF-1 and ErbB receptors [19]. Nevertheless, the relationship between MMP-2 activation and late MAPK phosphorylation observed in our Schwannoma cell model merits further investigation in primary Schwann cell culture.

In light of these findings, we conclude that extracellular UTP (which mimics nerve injury) stimulates Schwannoma cell migration and wound repair through a MMP-2-dependent mechanism via P2Y_2_ receptors and the activation of MAPK pathways. Moreover, evidence that P2Y_2_ receptors mediate the migratory response of Schwann cells evoked by PNS injury indicates that selective P2Y_2_ receptor agonists may provide new therapeutic opportunities for Schwann cell migration and peripheral nerve regeneration.

## Supporting Information

Figure S1 **Short hairpin RNA validation.** (**A**) RT4-D6P2T cells were transfected with shRNA directed against a non-targeting control (shRandom) or rat MMP-2 or P2Y_2_ genes, and after 48 h MMP-2 and P2Y_2_ mRNA levels were determined by quantitative RT-PCR. GAPDH was used as a constitutive gene. Statistical significance: *P≤0.05 compared to control cells. (**B**) RT4-D6P2T cells were transfected with shRandom, shMMP-2 or shP2Y_2_, and after 48 h MMP-2 and P2Y_2_ protein levels were determined by immunoblotting. GAPDH was used as a constitutive protein.
(TIF)

Figure S2 **Quantitative MMP-2 gene expression analysis.** Time–course MMP-2 gene expression analysis for Schwann cell line (A) and for primary Schwann cells (B) treated or untreated with UTP (250 µM). MMP-2 mRNA levels were determined by quantitative RT-PCR. GAPDH was used as a constitutive gene. Statistical significance: *P≤0.05, **P≤0.01, and *** *P*≤0.001.
(TIF)
