# Supplementary figures and images for: Uridine 5′-Triphosphate Promotes In Vitro Schwannoma Cell Migration through Matrix Metalloproteinase-2 Activation
Source: PLoS One. 2014 Jun 6;9(6):e98998. doi: 10.1371/journal.pone.0098998 (PMC4048211; doi:10.1371/journal.pone.0098998)

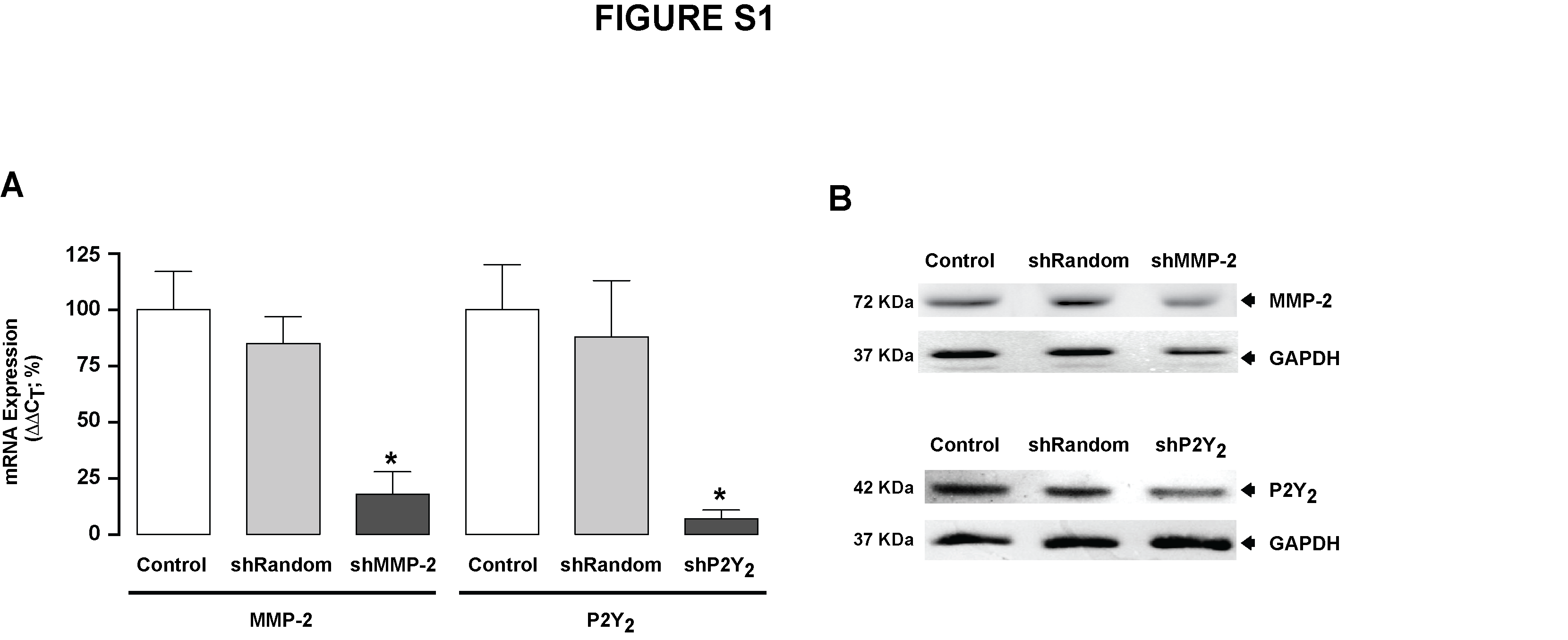

Supplement: Figure S1 — Short hairpin RNA validation. (A) RT4-D6P2T cells were transfected with shRNA directed against a non-targeting control (shRandom) or rat MMP-2 or P2Y2 genes, and after 48 h MMP-2 and P2Y2 mRNA levels were determined by quantitative RT-PCR. GAPDH was used as a constitutive gene. Statistical significance: *P≤0.05 compared to control cells. (B) RT4-D6P2T cells were transfected with shRandom, shMMP-2 or shP2Y2, and after 48 h MMP-2 and P2Y2 protein levels were determined by immunoblotting. GAPDH was used as a constitutive protein. (TIF) [file pone.0098998.s001.tif]

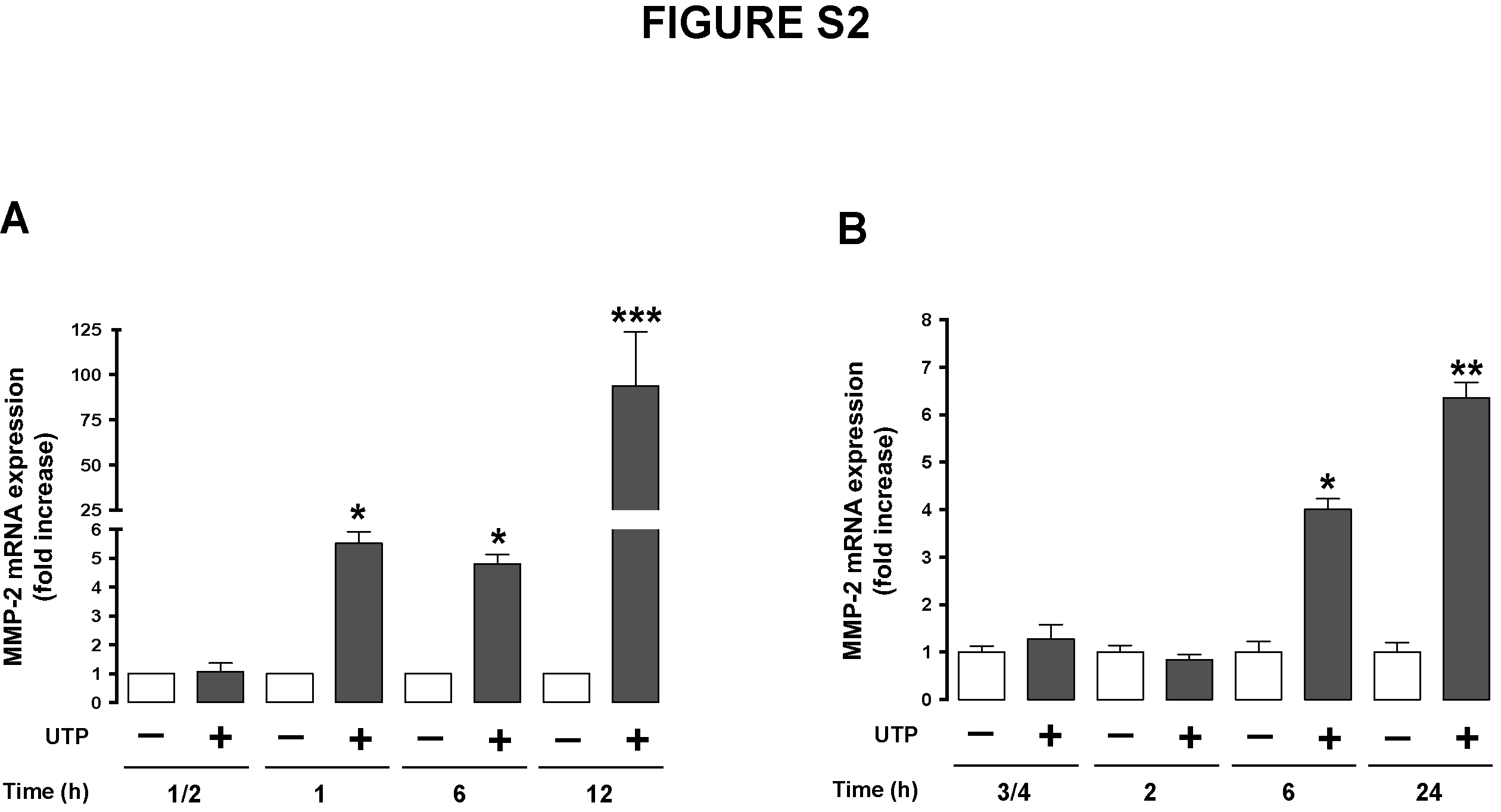

Supplement: Figure S2 — Quantitative MMP-2 gene expression analysis. Time–course MMP-2 gene expression analysis for Schwann cell line (A) and for primary Schwann cells (B) treated or untreated with UTP (250 µM). MMP-2 mRNA levels were determined by quantitative RT-PCR. GAPDH was used as a constitutive gene. Statistical significance: *P≤0.05, **P≤0.01, and *** P≤0.001. (TIF) [file pone.0098998.s002.tif]
